# Identification of Goat Supernumerary Teat Phenotype Using Wide-Genomic Copy Number Variants

**DOI:** 10.3390/ani14223252

**Published:** 2024-11-13

**Authors:** Lu Xu, Weiyi Zhang, Haoyuan Zhang, Xiuqin Yang, Simone Ceccobelli, Yongju Zhao, Guangxin E

**Affiliations:** 1 College of Animal Science and Technology, Southwest University, Chongqing 400715, China; lujiusym@163.com (L.X.); 2311508@tongji.edu.cn (W.Z.); swuzhanghy@163.com (H.Z.); zyongju@163.com (Y.Z.); 2 College of Animal Science and Technology, Northeast Agricultural University, Harbin 150030, China; xiuqinyang@neau.edu.cn; 3 Department of Agricultural, Food and Environmental Sciences, Università Politecnica delle Marche, 60131 Ancona, Italy; s.ceccobelli@staff.univpm.it

**Keywords:** goat, supernumerary teats/nipples, copy number variant

## Abstract

Supernumerary teats (SNTs) or nipples often emerge around the mammary line. This study performed a genome-wide selective sweep analysis (GWS) at the copy number variant (CNV) level using two selected signal calculation methods (*V*_ST_ and *F*_ST_) to identify candidate genes associated with SNTs in goats. A total of 12,310 CNVs were identified from 37 animals and 123 CNVs, with the top 1% *V*_ST_ values including 84 candidate genes (CDGs). Of these CDGs, minichromosome maintenance complex component 3, ectodysplasin A receptor associated via death domain, and cullin 5 demonstrated functions closely related to mammary gland development. In addition, 123 CNVs with the top 1% *F*_ST_ values were annotated to 97 CDGs. 5-Hydroxytryptamine receptor 2A, CCAAT/enhancer-binding protein alpha, and the polymeric immunoglobulin receptor affect colostrum secretion through multiple signaling pathways. Two genes, namely, RNA-binding motif protein 46 and β-1,3-galactosyltransferase 5, showed a close relation to mammary gland development. Six CNVs were identified and annotated to five genes by intersecting the top 1% of candidate CNVs with both parameters. These genes include *LOC102185621*, *LOC102190481*, and UDP-glucose pyrophosphorylase 2, which potentially affect the occurrence of BC through multiple biological processes, such as cell detoxification, glycogen synthesis, and phospholipid metabolism. In conclusion, we discovered numerous genes related to mammary development and breast cancer (BC) through a GWS, which suggests the mechanism of SNTs in goats and a certain association between mammary cancer and SNTs.

## Simple Summary

Supernumerary teats (SNTs) refer to developmental anomalies that occur during the embryonic period and are commonly found around the mammary line. We performed a genome-wide selective sweep analysis on 37 goats using copy number variants, and identified multiple genes involved in embryonic mammary gland development and the biological process of breast cancer. Our findings can be used as a reference for the further investigation of SNT phenotypic candidate genes.

## 1. Introduction

Supernumerary teats/nipples (SNTs) serve as indicators of reproductive performance and are a moderately heritable trait [1]. SNTs refer to extra nipples that appear along the mammary line [2]. SNTs in humans was reported as early as 1879 [3]. Supernumerary breast and aberrant breast tissue are considered ectopic breast tissues (EBTs), which carry the same risk of developing benign or malignant breast disease as a normal breast [4]. The presence of such EBTs has been detected in up to 6% of the population [5], and similar cases have been reported clinically [6,7,8,9]. SNTs can be due to familial inheritance, such as autosomal dominance, X-linked dominance, or recessive inheritance [10]. The early formation of the SNT phenotype results from the failure of the mammary spine to dissipate in a timely manner during embryonic development [11]. In the dairy industry, SNTs are considered epidermal abnormalities that negatively affect machine milking, udder health, and animal welfare [12]. This trait is a common abnormality in cows’ udders, in which it may be more influential. SNTs may increase the probability of bacterial infection, which can further lead to mastitis; meanwhile, excess nipples may be unsuitable for automated milking systems, which affects the economics of dairy farming [6,13]. SNT inheritance may be controlled by oligogenic or polygenic genes in cattle, with heritability estimates ranging from 0.09 to 0.63 [14]. In goats, SNTs are considered a heritable polygenic trait that occurs in several goat breeds [2,15,16]. The heritability of SNT varies considerably in different goat populations and is particularly common in high-milk-yielding breeds [2]. Considerable controversy surrounds the importance of SNT inheritance. Some studies have argued for the direct proportionality of the number of mammary glands to the lactation capacity of the udder. Goats with SNTs have more mammary parenchyma than those with double nipples [17]. However, extra nipples lack breast parenchyma and can negatively influence milk production [18] and the healthy growth of the lamb. Therefore, the genetic underpinnings of SNT and its functional importance remain to be fully elucidated. This study aimed to perform a genome-wide selective sweep analysis (GWS) to identify candidate genetic markers related to SNTs in goats using copy number variants (CNVs). The analysis results will aid in a further understanding of the hereditary basis of SNTs.

## 2. Materials and Methods

The Institutional Animal Care and Use Committee of Southwest University approved the experimental protocols (Permit no. IACUC-20210415-05). This work strictly adhered to international, national, and institutional animal ethical guidelines, and no animals were anesthetized nor euthanized during sampling. A total of 37 healthy female goats indigenous to Chongqing were sampled (8 Hechuan White goats and 29 Dazu black goats), 23 and 14 of which had supernumerary nipples (SNTs) and double nipples, respectively (Figure S1). Genomic DNA was extracted from the animals’ jugular venous blood using a TianGen blood DNA extraction kit DP304 (Tiangen, Beijing, China), in accordance with the manufacturer’s instructions. Whole-genome sequencing libraries were prepared in accordance with the TruSeq DNA sample preparation guide (Illumina, 15026486 Rev.C, San Diego, CA, USA). Sequencing was conducted using an Illumina NovaSeq 6000 platform (Annoroad Gene Technology, Beijing, China).

The raw data were filtered using Fastp (v0.20.0) [19], with the parameters “fastp -w 15 --cut_window_size 4 --cut_mean_quality 15 -5 3 -3 3”, to obtain high-quality reads (HQRs). BWA (v0.7.17) with the MEM algorithm was used to align the HQRs to the goat reference genome (GCA_001704415.1) [20]. Polymerase chain reaction repeats were removed using the Picard package (https://github.com/broadinstitute/picard, accessed on 19 October 2022) with the parameters of MarkDuplicates “MAX_FILE_HANDLES_FOR_READ_ENDS_MAP=8000”. CNVcaller was used to identify CNVs in all individuals with a silhouette coefficient ≤ 0.6 and a minor allele frequency ≤ 0.05 [21]. The optimal window size was 800 bp. A total of 23 female goats with the SNT phenotype were used as cases and 14 female goats with the normal phenotype (double papillary) were used as controls for the *F*_ST_ [22] and *V*_ST_ [23] calculations. The *F*_ST_ and *V*_ST_ values of each CNV (accounting for the top 1%) were calculated using VCFtools (v0.1.16) [24] and a Perl script, respectively. A Kyoto Encyclopedia of Genes and Genomes (KEGG) functional annotation of the candidate genes (CDGs) from high-signal CNVs was performed using KOBAS (http://bioinfo.org/kobas/genelist/, accessed on 13 May 2024) with a corrected *p* value ≤ 0.05 and the homo sapiens gene set, which indicates significantly enriched pathways/terms.

## 3. Results and Discussion

**Figure 1:**
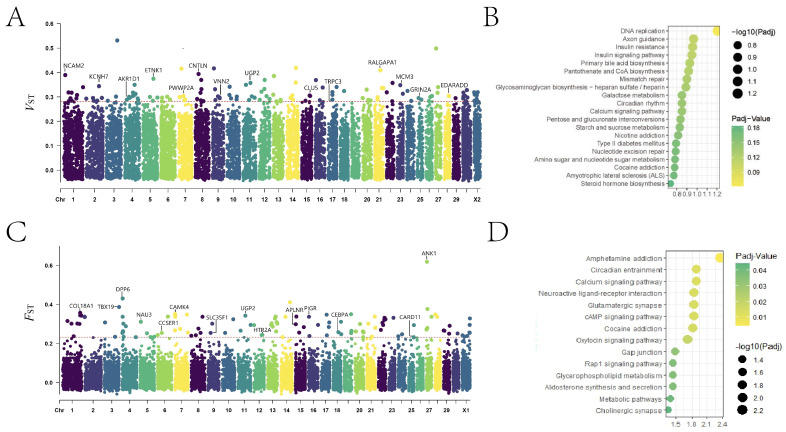
(**A**) A Manhattan map of the wide-genomic sweep analysis of the goat supernumerary teat phenotype using *V*_ST_. (**B**) The top 20 KEGG pathways enriched by candidate genes from CNVs with the top 1% *V*_ST_ values. (**C**) A Manhattan map of the wide-genomic sweep analysis of the goat supernumerary teat phenotype using *F*_ST_. (**D**) The 14 KEGG pathways significantly enriched by the candidate genes from CNVs with the top 1% *F*_ST_ values.

A total of 12,310 CNVs were identified across all autosomes and the X chromosome (CHR) of all animals. Specifically, 123 candidate CNVs with the top 1% *V*_ST_ values (*V*_ST_ ≥ 0.2840375) were initially obtained (Figure 1A). The CNV with the highest signal (*V*_ST_ = 0.531194081) was located on CHR3 (86,842,501–86,864,500 bp). The 123 CNVs included 84 CDGs. The KEGG results reveal the enrichment of 27 CDGs in 54 KEGG pathways (Figure 1B). The majority of genes exhibited enrichment in pathways belonging to biosynthesis, metabolism, and cell proliferation. Notably, 3 of the 27 CDGs, namely minichromosome maintenance complex component 3 (*MCM3*) (Table S4), ectodysplasin (EDA) A receptor associated via death domain (*EDARADD*), and cullin 5 (*Cul5*), were associated with breast development.

During mammary gland development, rapid tissue remodeling occurs with epithelial invasion into the stroma, whereas an acute phase response transpires during mammary gland degeneration in mice [25]. Many scholars assume that the inflammatory response and processes during this stage are comparable to changes in cancer [26,27]. *MCM3*, which is localized to mammary epithelial cells, is specifically expressed during mammary degeneration, and it promotes glandular cell regeneration [28]. Moreover, experiments have confirmed the involvement of *MCM3* in the transformation of human breast epithelial cells [29] and have demonstrated that it contributes to breast development [28].

The key gene *EDARADD* in the EDA pathway profoundly influences the development of ectoderm-derived structures, including sweat and mammary glands [30]. The adaptor protein Edaradd, together with Eda and its receptor Edar, constitutes the EDA signaling pathway [31]. The activation of the EDA pathway can induce nuclear factor-κB-mediated transcription, which is involved in the formation and morphology of embryonic mammary glands [32]. Two types of ectodermal dysplasia (ED) are associated with sweat glands in human clinical cases: the hypohidrotic type and hidrotic ectodermal dysplasia [33]. According to a study on a certain population, more than 30% of male X-linked hypohidrotic ectodermal dysplasia patients had missing, simple, or multiple nipples [34]. Moreover, some female carriers experienced recurrent chest infections, and 80% of the 24 mothers interviewed reported insufficient milk production when feeding their infants [34]. Another work found a missense mutation in the *EDARADD* gene at Pro153Ser of rat Chr7 [35]. Mice with homozygous variants of the *EDARADD* gene produced a similar ED phenotype, and female mice were unable to feed their offspring due to mammary gland defects [35,36,37]. Furthermore, the EDA and Wnt/β-catenin pathways exert a synergistic effect on the formation of the mammary plate during the embryonic period [32]. *Cul5* serves as a positive regulator of the proliferation of HC11 cells (a mouse mammary epithelial cell line), which mediates the stimulation of mRNA expression by Tau and the subsequent phosphorylation of mammalian target of rapamycin proteins; this condition intricately regulates mammary gland development [38].

This study also identified 123 CNVs with *F*_ST_ values in the top 1% (*F*_ST_ ≥ 0.231683, Figure 1C). The CNV with the highest signal (*F*_ST_ = 0.618451) was located on CHR27 (4,664,501~4,667,500 bp). Of these CNVs, 11 were detected on CHR4 and 1 on CHR23 and CHR X2. A total of 97 CDGs were annotated to these CNVs. Among these CDGs, 40 exhibited enrichment in 127 KEGG pathways, and significant enrichment was observed in 14 pathways (Figure 1D). Furthermore, six CDGs displayed an association with lactation. Three of these genes, 5-hydroxytryptamine receptor 2A (*HTR2A*), CCAAT enhancer binding protein alpha (*CEBPA*), and polymeric immunoglobulin receptor (*pIgR*), showed a close linkage to the biological function of breast epithelial cells. Moreover, two genes, namely RNA-binding motif protein 46 (*RBM46*) and β-1,3-galactosyltransferase 5 (*B3GALT5*), are potentially related to mammary gland development.

The action of serotonin (5-HT), a neurotransmitter produced in mammary epithelial cells (MECs), regulates milk secretion in various species [39]. Five 5-HT receptors (5-HTR) show expression in small mammary vessels, whereas MECs express *HTR2A* [40]. *APLNR* displays an association with colostrum secretion, and its expression gradually increases with mammary gland development during pregnancy [41]. *ANK1* in dairy cows exerts a regulatory effect on mammary gland development [42]. CEBPs display differential expression throughout mammary gland development and can bind to the *CSN2* promoter to regulate its expression [43]. *CEBPA*, the first member of this family to be identified, is subject to regulation by lactogenic hormones in MECs [44]. Meanwhile, *CEBPA,* which affects milk fat formation, is regulated by *AGPAT6* [45]. The *PIGR* gene serves as a polyimmunoglobulin receptor; it is remarkably upregulated during lactation and mediates IgA transport in MECs, which affects colostrum secretion in mice [46].

*RBM46* is an RNA-binding protein of unknown function. Mammary gland differentiation initiates in the embryonic ectoderm [47]. The knockout of *RBM46* leads to the downregulation of most trophoblast ectodermal markers in mouse embryonic stem cells, which prevent the distribution of blastomere cells to the trophoblast ectoderm in mouse embryos [48]. Thus, we speculated that *RBM46* may indirectly regulate breast tissue development. The *B3GALT5* gene encodes β1,3-galactosyltransferase 5 (β3Gal-T5) and contributes to the synthesis of type 1 Lewis antigens, which are well-known tumor markers [49]. The knockout of *B3GALT5* does not affect normal cells but can result in cancer-specific apoptosis [50]. The upregulation of *B3GALT5* promotes the expression of β-catenin and epithelial-to-mesenchymal transition (EMT) activator zinc finger transcription factors in breast cancer (BC) stem cells and regulates EMT, cell migration, and mammosphere formation [51]. Studies on papillary morphological differentiation have shown that during embryonic mammary development, mammary epithelial cells interact with mesenchymal cells to form the dense mammary mesenchyme and induce the specialization of epithelial cells for nipple formation [52]. EMT is a necessary process of mammary gland formation. If epithelial cells lack cell polarity and lose their connection with the basement membrane and other epithelia, they will transform into the mesenchymal phenotype, which results in the cells showing high migration and invasion, anti-apoptosis, and degradation of the extracellular matrix; such a condition leads to cancer metastasis and invasion [53]. In humans, multiple nipples and breasts serve as markers of abnormal development, which pose the potential threat of malignant disease. They are often associated with BC, which is a heterogeneous disease. Basal-like BC (BLBC) is one of the four subtypes of BC [54]. BLBC cells show susceptibility to EMT, which is likely to trigger tumor cell metastasis [55]. These properties of BLBC exhibit a potential relation to the breast genesis gene *B3GALT5* detected in this study. In conclusion, *B3GALT5* is closely related to mammary gland formation and the upstream physiological changes in the embryonic papillary formation process.

**Figure 2:**
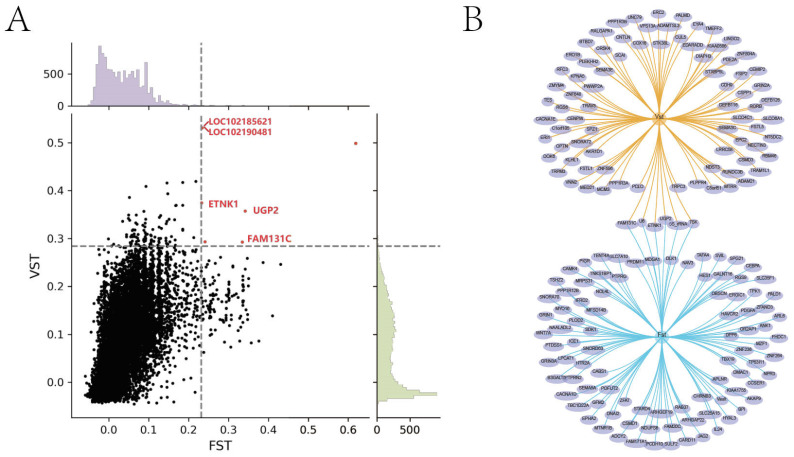
(**A**) Intersection of top 1% CNVs between *V*_ST_ and *F*_ST_. (**B**) Intersection map of *V*_ST_ and *F*_ST_ in terms of top 1% CNV annotated genes.

Six interacting CNVs were obtained from those with the top 1% *F*_ST_ and *V*_ST_ values (Figure 2A), and five CDGs, family with sequence similarity 131 member C (*FAM131C*), *LOC102185621*, *LOC102190481*, UDP-glucose pyrophosphorylase 2 (*UGP2*), and *ETNK1*, were annotated. All of them are related to BC, except for *FAM131C*.

Glutathione S-transferase M1 (GSTM1), which is encoded by *LOC102185621* and *LOC102190481*, is an important detoxification enzyme. It plays a crucial role in the electrophilic coupling reaction and in the maintenance of the balanced redox state of cells [56,57]. GSTM1 is markedly associated with BC. In particular, the loss of function of GSTM1 deprives cells of the ability to perform detoxification, which results in DNA damage [58]. Therefore, females with *GSTM1* gene deletion are at an increased risk of cancer development [59]. A previous study suggested that *UGP2* regulates the N-glycosylation modification of a range of proteins, including the epidermal growth factor receptor (EGFR) [60]. The lack of EGFR serves as a clinical indicator of basal BC [61]. *UGP2* shows an association with the occurrence of various cancers and influences the proliferation and migration of cancer cells [62,63]. Moreover, *ETNK1* participates in the main metabolic pathway of phosphatidylethanolamine (PE) [64]. The level of PE is higher in BC cells than in normal breast cells, and *ETNK1* serves as the primary cause of the increase in PE level in cancer cells [65].

In regard to human medicine, EBT is more likely to become cancerous than normal breast tissue and thus must be screened and prophylactically removed; some evidence shows that although EBT may develop benign or malignant lesions, its occurrence is rare [66]. For confirmation, if signs of malignancy develop after the diagnosis of EBT, the usual standard BC treatment procedures must be followed [66]. In addition, SNTs account for the developmental origin of 0.6% of BCs [67], and accessory mammary glands may develop mastitis [68]. Possibly a result of their shorter lifespans than humans, the incidence of breast tumors in most small ruminants is extremely low, which causes difficulty in the quantification of their incidence, which is usually limited to single case reports [69,70,71]. Although neoplastic lesions in the goat mammary gland are rare, considering that poor breast architecture may increase the risk of bacterial infection in the mammary glands, which causes breast lesions and mastitis [72], these intersections may play a potential role in the development of BC in SNT goats.

This study identified that the genes related to the SNT phenotype are not only related to breast development and lactation function but also contribute to the probability of BC development. Although evidence supporting the correlation between SNT and BC in goats is lacking, we hypothesized that a large number of genes and pathways involved in BC participate in the development of breast abnormalities. In addition, the identification of a large number of genes related to breast development and lactation indicates the possible relation of SNT to the lactation capability and milk quality of animals. Further observations and research are necessary to confirm these connections.

## 4. Conclusions

In this study, we used a CNV dataset based on GWS to describe the genes (*MCM3*, *EDARADD*, *Cul5*, *RBM46*, and *B3GALT5*) that may be associated with the embryonic SNT phenotype and four genes (*LOC102185621*, *LOC102190481*, *UGP2* and *ETNK1*) associated with BC. These genes may participate in the formation of SNTs and are closely related to the subsequent development of SNTs into BC. In general, we screened specific CNVs associated with SNT traits, and screened out related candidate genes. This work provides a reference for the further study of SNT phenotypic candidate genes in goats.

## Data Availability

Genome sequencing data from 37 individuals were uploaded to the Sequence Read Archives of the National Center for Biotechnology Information (PRJNA1032158, PRJNA732249, and PRJNA734742; Table S1), and the variation data of all individuals were uploaded to the China National Center for Bioinformation database (GVM000711).

## Supplementary Materials

The following supporting information can be downloaded at https://www.mdpi.com/article/10.3390/ani14223252/s1, Figure S1: Nipple pictures of SNTs and double nipples; Table S1: Basic information of 37 individual goats; Table S2: The top 1% of candidate CNV obtained by *V*_ST_; Table S3: Kyoto Encyclopedia of Genes and Genomes (KEGG) analysis of candidate genes from the CNVs with the top 1% of *V*_ST_ values; Table S4: Full name for the gene abbreviation in discussion; Table S5: The top 1% of candidate CNV obtained by *F*_ST_; Table S6: Kyoto Encyclopedia of Genes and Genomes (KEGG) analysis of candidate genes from the CNVs with the top 1% of *F*_ST_ values; Table S7: Genes annotated by intersection of CNV obtained by *F*_ST_ and *V*_ST_.
