# Supplementary figures and images for: Identification of Goat Supernumerary Teat Phenotype Using Wide-Genomic Copy Number Variants
Source: Animals (Basel). 2024 Nov 13;14(22):3252. doi: 10.3390/ani14223252 (PMC11591440; doi:10.3390/ani14223252)

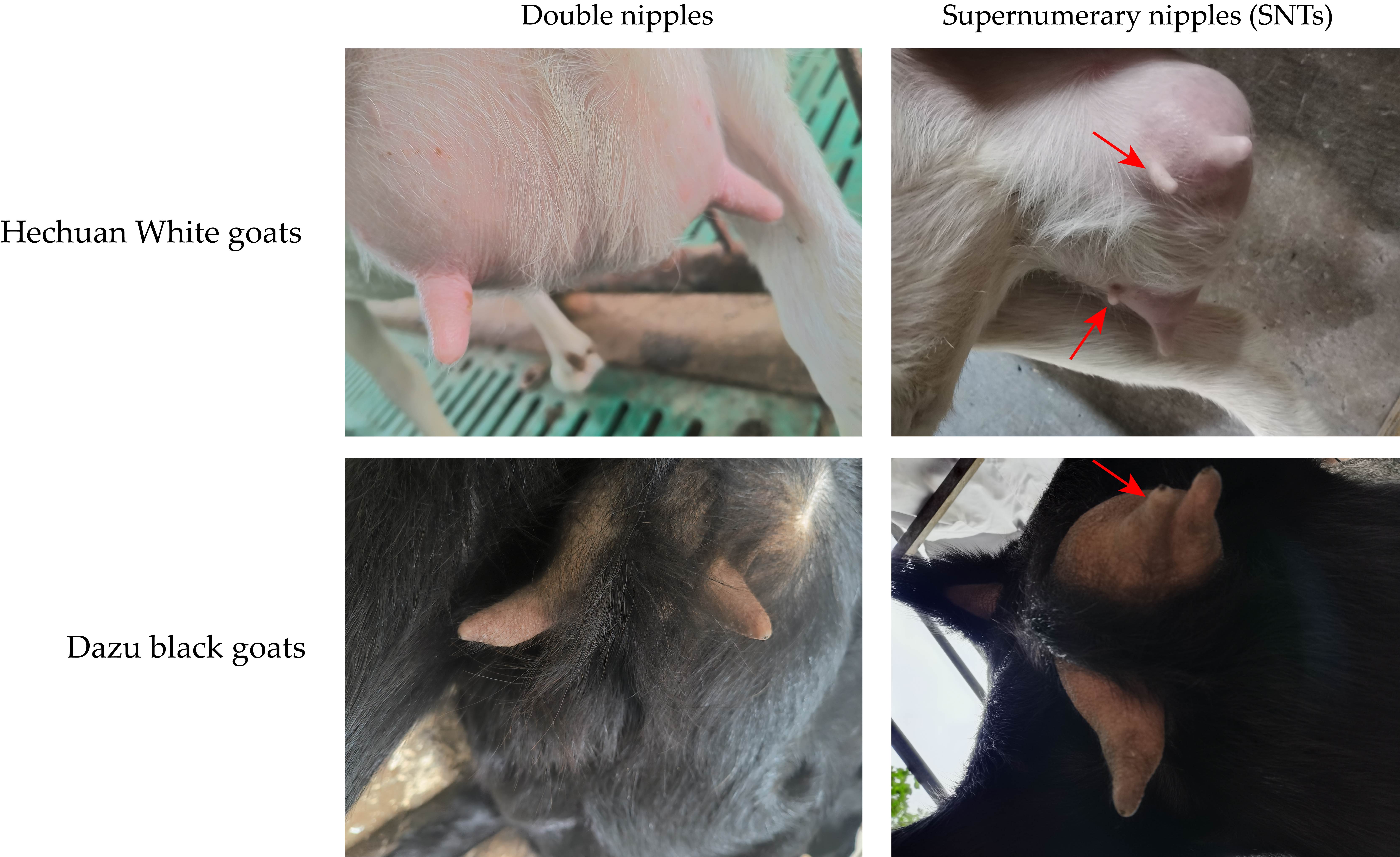

Supplement: Supplementary file 1 [file animals-14-03252-s001.zip › Figure S1.jpg]
